# Supplementary material for: Association between oral microbiome and seven types of cancers in East Asian population: a two-sample Mendelian randomization analysis
Source: Front Mol Biosci. 2023 Nov 21;10:1327893. doi: 10.3389/fmolb.2023.1327893 (PMC10702768; doi:10.3389/fmolb.2023.1327893)
Supplement: Supplementary file 1 [file DataSheet1.ZIP › Supplementary Materials/MR plots of saliva/saliva═╝/colorectal cancer/pheno.1211_to_colorectal cancer_leave_one_out.pdf]

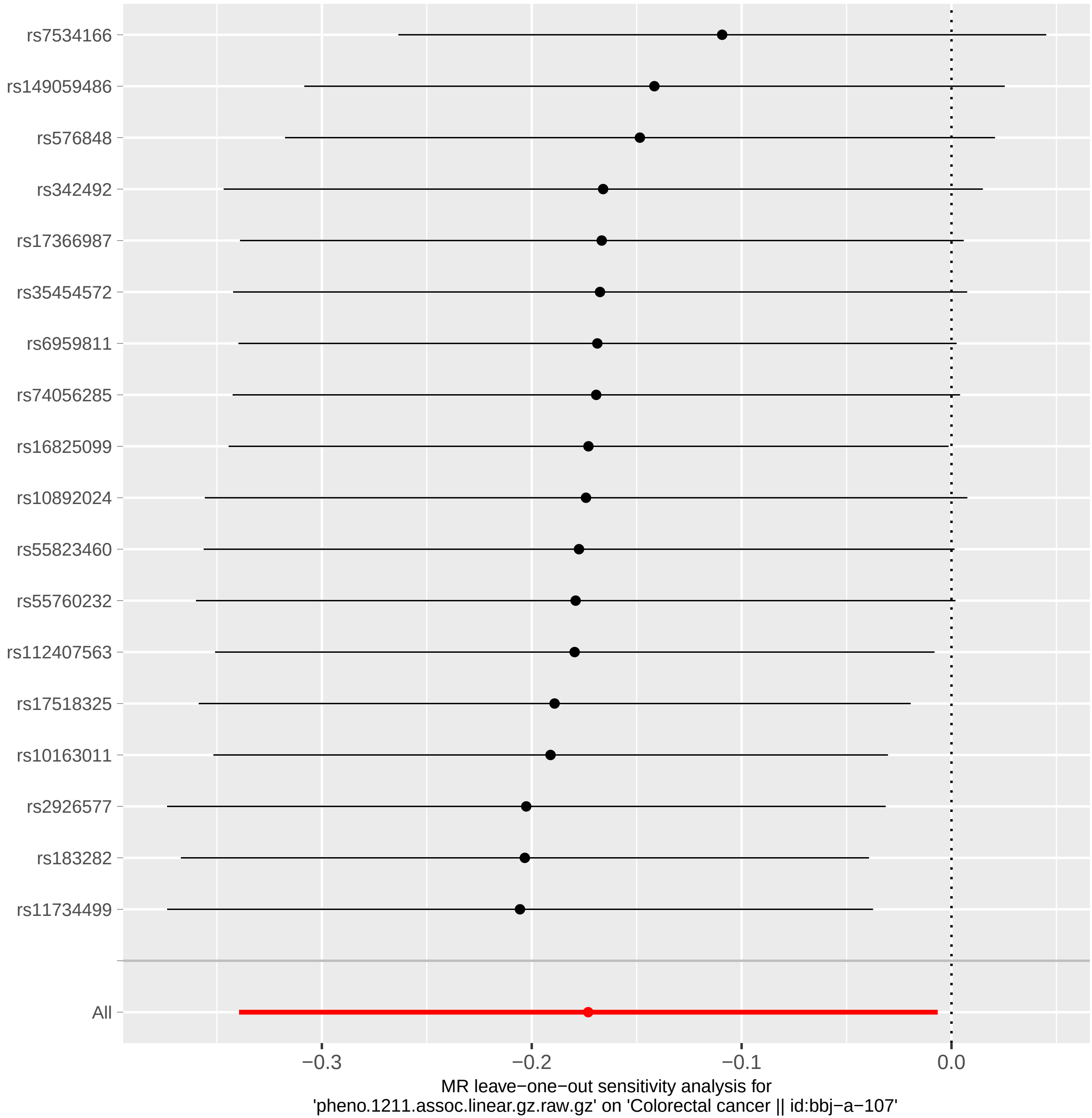
rs7534166
rs149059486
rs576848
rs342492
rs17366987
rs35454572
rs6959811
rs74056285
rs16825099
rs10892024
rs55823460
rs55760232
rs112407563
rs17518325
rs10163011
rs2926577
rs183282
rs11734499
All
−0.3
−0.2
−0.1
0.0
MR leave−one−out sensitivity analysis for
'pheno.1211.assoc.linear.gz.raw.gz' on 'Colorectal cancer || id:bbj−a−107'
